# Incorporating neglected non-communicable diseases into the national health program—A review

**DOI:** 10.3389/fpubh.2022.1093170

**Published:** 2023-01-10

**Authors:** Sudip Bhattacharya, Petra Heidler, Saurabh Varshney

**Affiliations:** ^1^Department of Community and Family Medicine, All India Institute of Medical Sciences, Deoghar, Jharkhand, India; ^2^Department for Economy and Health, University for Continuing Education Krems, Krems an der Donau, Austria; ^3^Department of International Business and Export Management, IMC University of Applied Sciences Krems, Krems an der Donau, Austria; ^4^Department of Health Sciences, St. Pölten University of Applied Sciences, Sankt Pölten, Austria; ^5^Department of ENT (Otorhinolaryngology), All India Institute of Medical Sciences, Deoghar (AIIMS Deoghar), Deoghar, India

**Keywords:** non-communicable disease (NCD), diabetes, hypertension, NCD and risk factors, NCD burden, neglected disease initiative, neglected NCD

## Abstract

Poor nations are already facing the heat of double burden of communicable and non-communicable diseases (NCDs), often known as chronic illnesses, which are characterized by a protracted course and are multifactorial in causation. In addition to this, neglected non-communicable diseases (NNCD) in the form of gout, sickle cell disease, accidents and many more are likely to be one of the biggest public health challenges soon. Nearly three-quarters (31.4 million) of all NCD-related fatalities occur in developing nations. In terms of morbidity and mortality, the “BIG FOUR” NCDs—diabetes, cancer, chronic respiratory diseases, and cardiovascular diseases—are widely acknowledged as the main contributors to global health loss. However, other NCDs account for 55% of the global burden of NCDs and are frequently neglected in terms of premature death, increased Disability Adjusted Life Years (DALY), and decreased Quality-Adjusted Life Year (QALY). We have briefly discussed the disease burden of a few significant, yet neglected NCDs in this paper.

## Introduction

### Global and national burden of NCDs

Non-communicable diseases (NCDs), usually referred to as chronic diseases, are brought on by a confluence of physiological, behavioral, physiological, environmental, and environmental factors. Annually, they claim the lives of 41 million people, or 71% of all fatalities globally. The NCDs claim more than 15 million lives each year in people between the ages of 30 and 69 with low- and middle-income countries account for 85% of these “premature” deaths. Low- and middle-income nations represent 77% of all deaths from NCDs (1). Cardiovascular diseases (which kill 17.9 million people annually), cancer (9.3 million), respiratory problems (4.1 million), and diabetes (1.5 million) round out the top five causes of NCD deaths (2). Global Framework action on prevention and control of NCDs keeps into consideration the lifestyle risk factors such as nutrition, physical inactivity, alcohol and tobacco use, and related comorbidities such as hypertension, obesity, high cholesterol level, diabetes, etc. (2). Non-communicable diseases (NCDs) account for a sizable portion of the global disease burden and have a significant economic impact, particularly in nations with low and moderate incomes. According to World Health Organization, the top three fatalities in 2019 were ischemic heart disease, stroke, and chronic obstructive pulmonary disease (COPD), all of which are NCDs. More than 23 million people died from NCDs (2), which were also six of the top 10 global causes of death in 2019. Almost 36 million people who die from all NCDs each year are represented by this (equivalent to 63 per cent of global deaths). Between the ages of 30 and 70, 14 million individuals die prematurely globally, with 86 percent occurring in low- and middle-income nations (3). Six of the top 10 diseases in 2019 with the most disability-adjusted life years (DALYs) were NCDs. From 1990 to 2019 for all ages, these NCDs rose on the list of the top causes of DALYs. In 2019, NCDs accounted for seven of the top 10 major causes of DALY among the 25- to 49-year-old age group, which is most productive age group. The sum of the DALYs for this age group caused by these seven disorders accounted for one-fourth of all DALYs during that year. The first nine major causes of DALYs for people aged 50–74 in 2019 were all NCDs, accounting for 45% of all DALYs for this age group.

In India, the burden of non-communicable diseases has increased over the past 20 years, replacing infectious diseases, under nutrition, maternal disorders, and pediatric illnesses (NCDs). In 2019, NCDs were responsible for 65% of all fatalities in the nation (4). The Indian government approved the global NCD monitoring targets in 2013 and developed the country's own NCD targets (10) and indicators (21) that must be met by 2025 (4, 5). Every region in India is vast and unique in terms of its socio-demographic profile, disease epidemiology, cultural and lifestyle traditions, and economic and social development (6). Despite of the economic and health system profiles of the various regions, NCDs and the risk factors associated with them are common and becoming a major public health concern.

### Consequences of NCDs

Non-communicable diseases (NCDs) have a significant economic impact on populations in addition to causing morbidity and mortality. The expansion of the national economy as well as personal financial security is negatively impacted by non-communicable diseases (NCDs). In low- and middle-income countries, NCDs frequently have an impact on a person's most productive years of life. People with NCDs must pay high healthcare costs and have few job possibilities, which put households at greater risk financially. Increase in healthcare expenditure, and decrease in productivity put strain on the economy of developing countries and impedes social and economic advancement. It is crucial to discuss the social and economic repercussions of the “chronic emergency” of NCDs in India because India contributes significantly to global mortality and lost disability adjusted life years (DALYs) (7).

With high rates of NCD morbidity and mortality at a time when communicable diseases are not yet under control, India is one of the developing countries that has gone through a “double burden” epidemiological transition. At a time when communicable diseases had fallen to noticeably lower levels, chronic conditions have seen a rise in most developed countries. Secondly, different states in India are at various stages of the transition. This epidemiological transition is being driven by a change in demographics. In 2025, there will be 7.6 percent more people 65 who are 65 years and above than there were in 2000 (8).

## What are neglected non-communicable diseases (NNCD)?

**Figure 1:**
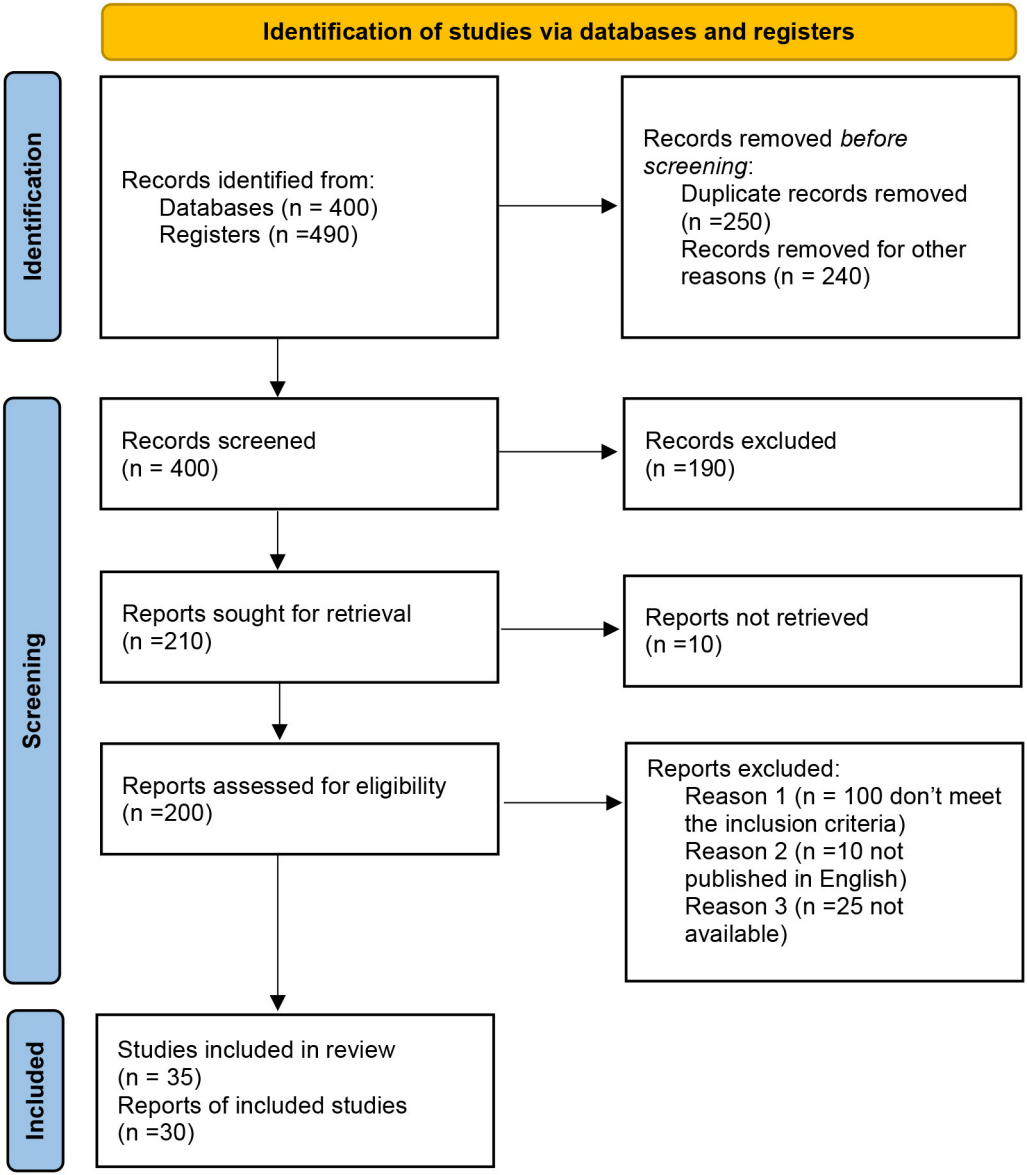
Identification of studies.

While diabetes, chronic obstructive pulmonary disease (COPD), cancer, and cardiovascular diseases all contribute significantly to global health loss, other NCDs account for a greater portion (55%) of the global NCD burden. Musculoskeletal conditions, particularly low back and neck pain, depression, substance abuse disorders, liver cirrhosis, chronic kidney disease, asthma, various digestive conditions (peptic ulcer, anxiety disorders, congenital anomalies, and hemoglobinopathies) are a few of the more serious ones. Contrary to the “big four” NCDs, many of these illnesses result in chronic disability rather than early mortality, which suggests that preventing chronic disability should be a top priority for any health system (Figure 1) (9). The current study aims to estimate the global burden of NNCDs and to find out the possible ways to address this within National Programme for Prevention and Control of Cancer, Diabetes, Cardiovascular Diseases and Stroke (NPCDCS) in India.

## Methodology

We searched the databases “*MEDLINE, PsycINFO, Scopus, Web of Science, and Google scholar*” using the following terms: (“diabetes”) and (“diabetes mellitus”), (“type 1 diabetes”), (“nutritional programs in India”), (“Health policies in India”) (“juvenile diabetes in India”), NCDs, NNCDs, hypertension, diabetes, cancer, gout, sickle cell disease, etc. This review includes all pertinent publications and policy papers (MOHFW, INDIA) available in the public domain from 2005 to support its claim. All relevant articles were included and a total of 890 articles were extracted after database searching. Initially, 250 duplicated information articles and 240 records were removed. By screening the article's titles and abstracts, 190 irrelevant articles were removed, and 10 reports were not retrieved, 135 articles were excluded after reviewing the full-text articles due to not fulfilling the research question and the review inclusion criteria. Finally, 35 studies and 30 reports met the inclusion criteria and entered the research process of the review.

## Results and discussion

The neglected non-communicable diseases include gout, sickle cell disease, asthma, chronic kidney disease, liver cirrhosis, alzheimer's disease and many more which are not even recognized.

### Gout

Gout, the most common kind of inflammatory arthritis, is caused by an accumulation of uric acid crystals in and around the joints as a result of hyperuricemia. The range of gout prevalence and incidence vary differently from 1 to 6.8% and 0.58 to 2.89 per 1,000 person-years, respectively. It affects more men than women as they become older and in specific ethnic communities. Obesity, nutritional variables, and other conditions are only a few of the many risk factors for gout. It has been observed that gout has connections with concomitant conditions such sexual dysfunction, tachycardia, obstructive sleep apnea, and osteoporosis, and deep vein thrombosis. In people with gout, specific patterns of comorbidity clustering (in the form of heart disease, chronic kidney disease) have been identified (10).

The prevalence of gout in India is unclear. According to a study conducted by the Community Oriented Program for Control of Rheumatic Diseases (ILAR COPCORD) in the Indian village of Bhigwan, the prevalence of gout is 0.12% (11). According to a study from Vellore, 15.8% of the patients are under 30 years old; urban Indians are more affected than rural Indians are, and because metabolic syndrome is more prevalent in younger people, their first gout attack usually happens 10 years earlier (12). Another Indian investigation revealed that the metabolic syndrome's laboratory and anthropometric indicators are linked to elevated uric acid levels (13).

### Sickle cell disease

Sickle Cell Disease (SCD) has been recognized as a global issue of public health concern (World Health Organization and United Nations). Excess mortality in children under five is a result of the high frequency of untreated non-communicable illnesses, particularly SCD.

In Nigeria, India, and the Democratic Republic of the Congo, where the disease affects up to 2% of the population and the transmission prevalence rate (sickle cell trait) is as high as 10% to 30% (14). At least 150,000 infants are thought to be born each year with SCD in Nigeria alone. Sickle hemoglobin has been found in the tribal populations of the Nilgiri Hills (15), Bihar (16) and Odhisa (17). The sickle cell gene has been found to be common among several population groups, including the scheduled tribes, scheduled castes, and other backward classes in India, which are three socioeconomically disadvantaged ethnic groups (17–20). There have been reports of some startling discrepancies, such as a tribal community in Valsad having lesser sickness as compared to non-tribal population in Nagpur (21), which is attributed to the exceptionally high alpha thalassemia rates in the tribal group. The apparent difference in disease severity among numerous sources in India is partially attributable to the method of patient assessment (22, 23), with studies of hospitalized cases reported severe disease in contrast to the Burla (24) where mild to moderate diseases have been reported from out patient department. In a different study performed in Sindh Province, sickle cell disease patients were identified to have sickle—cell disease in 40% of cases, and of the 54% who had SS disease, only 16% had alpha thalassemia (25), compared to over 50% and 86% observed in Odisha (21) and Valsad (21), respectively.

### Chronic kidney diseases

Chronic kidney diseases (CKD) are commonly found among the individuals having diabetes and high blood pressure (26). The global burden of morbidity and death is directly impacted by CKD through its impact on cardiovascular risk and ESKD (27). Globally, there are 4.902–7.083 million individuals with end-stage kidney disease (ESKD) who need renal replacement therapy, while the prevalence of chronic kidney disease is 13.4% (11.7–15.1%).

The reported prevalence of CKD varies by area and ranges from 1 to 13%; most recently, statistics from the Kidney Disease Data Center Study of the International Society of Nephrology revealed a prevalence of 17% (28). There are many different etiologies of CKD in India. Parts of the states of Andhra Pradesh, Odisha, and Goa have high rates of CKD of unidentified etiology (CKDu), a chronic multifocal nephritis with a covert beginning and sluggish progression (29).

The percentage of deaths in India attributed to kidney failure increased by 38% between 2001–2003 and 2010–2013. From 0.59 million fatalities in 1990 to 1.18 million deaths in 2016, CKD was the major cause of deaths in India. As reported by the Million Death Study, there were 136,000 renal failure-related deaths in 2015. According to an estimate from 2018, there are ~175,000 chronic dialysis patients in India, translating to a prevalence of 129 per million people. According to a systematic review, about two-thirds of patients with kidney failure were projected to passed away in 2010 without obtaining dialysis facility (30). In another study, total 5588 subjects were analyzed. The mean ± SD age of all participants was 45.22 ± 15.2 years (range 18–98 years) and 55.1% of them were males and 44.9% were females. The overall prevalence of CKD in the SEEK-India cohort was 17.2% with a mean eGFR of 84.27 ± 76.46 versus 116.94 ± 44.65 mL/min/1.73 m^2^ in non-CKD group while 79.5% in the CKD group had proteinuria. Prevalence of CKD stages 1, 2, 3, 4 and 5 was 7%, 4.3%, 4.3%, 0.8% and 0.8%, respectively (31–33).

### Liver cirrhosis

Liver disease is among the leading causes of death and morbidity all over the world. It was the 11th main cause of mortality and 15th significant cause of morbidity in the world in 2016, accounting for 2.2% of deaths and 2.2% of disability-adjusted life years. In 2017, 1.32 million people died from CLD, with roughly two thirds being men and one third being women (34). Liver cirrhosis is a significant health issue in India as well. India accounted for one-fifth (18.3%) of all fatalities due to cirrhosis globally, with 259,749 liver disease deaths recorded there in 2017, or 2.95% of all deaths (35). India alone was responsible for 18.3% of the two million deaths caused by liver disease worldwide in 2015. India bears a heavy burden of liver disease and 2.1% of all fatalities in India in 2016 were attributed to various CLDs and cirrhosis (36). Recent evidence suggests that the prevalence of metabolic syndrome among adults in India is 30% (95%CI: 28–33%). Although, they were unable to identify any significant geographic or temporal (from 2003 to 2019) pattern. The authors documented a much higher incidence of liver disease among population having advanced age, in facility based studies, in urban areas and among females (37–41). A prevalence of NAFLD between 19 and 32.0% was reported in the few population-based studies. Another population survey from Kerala then revealed that both urban (55.2%) and rural (43.4%) people were suffered from CLD (42).

### Alzheimer's and dementias

Alzheimer's disease and other cognitive disorders constitute a serious and expanding burden to global health, with 40–50 million people suffering from dementia. Dementia prevalence in South Asia was 1.9% in 2005; by 2020 and 2040, it is expected to reach 3.6 million and 7.5 million people, respectively. Numerous epidemiological studies have found that the prevalence of dementia in India ranges from 2 to 35 per 1,000 individuals (43, 44). The likelihood of developing dementia rises with age as an example, dementia affects around 20% of adults over 80 years of age. The average age of presentation in India is 66.3 years, which is about 10 years younger than the average age in wealthy nations (44). In a study from rural India, a prevalence rate of 0.84% (95% CI, 0.61–1.13) and 1.36% (95% CI, 0.96–1.88) were found for all forms of dementia with a CDR score of at least 0.5 in the population of people aged 55 years and older, 65 years and older, respectively. The prevalence of AD was 0.62% (95% CI: 0.43–0.88) in the population of adults 55 and older, and 1.07% (95% CI: 0.72–1.53) in the population of adults 65 and older. Although neither gender nor education were associated with prevalence, the occurrence of AD and all other dementias was significantly connected with older age. The results indicated that elderly rural persons had a higher incidence of dementia. Compared to the senior population living in urban regions (20 per 1,000; 95% CI: 0.01–0.03), which has a rate of (30 per 1,000; 95% CI: 0.01–0.05) (45). According to a dementia research in India, it is expected to affect more than 14 million Indians by the year 2050.

According to the Global Burden of Disease report, which was published on Lancet Public Health, India is predicted to have 11.44 million dementia sufferers by the year 2050, up from 3.84 million in 2019. According to the analysis, population aging, and growth will account for the majority of the 197% increase in dementia cases, but other variables like as smoking, obesity, high blood sugar, and a lack of awareness of the condition will also be important (46).

### Asthma

In patients with asthma it becomes more difficult to exhale. It is a chronic disease that affects the tubes that carry air into and out of lungs. According to recent estimates, there are more than 339 million people diagnosed with asthma globally (47). Usually, asthma runs in families. If one or both of one's parents have asthma, the likelihood of developing it is increased (48).

India is the asthma capital of the world since it accounts for almost 42% of all associated deaths and represents 11.1% of the global burden of asthma (49). According to the worldwide asthma report 2018, 62.2% of adults and 6% of children living in India have asthma (50). 13.1% of teenagers (*n* = 121) had bronchial asthma, of which 10.3% experienced attacks within the previous year. Males (8.77%) had a greater prevalence rate than females (4.33%) (51). As per INSEARCH study, asthma prevalence in the nation is 2.05%, with 17.23 million people are affected (52). The most recent Worldwide Burden of Disease report (GBD, 1990-2019) predicts that 34.3 million Indians will have asthma, accounting for 13.09% of the global burden. Additionally, asthma was responsible for 13.2 deaths per 1,000 individuals in India (53).

India has lower rates of asthma prevalence (5–15%) than do European nations (>20%), but the condition is more severe (>40% of cases), less frequently diagnosed, and undertreated. India's rural areas have 3.6 lakh more severe asthmatics than its metropolitan areas (1.6 lakh) (54).

### Alcohol and substance abuse

According to the most recent estimates, 13% of all drug users, or 36.3 million people, take illicit drugs. 5.5% of persons worldwide between the ages of 15 and 64 had taken drugs at least once in the previous year. Alcohol abuse causes 3 million deaths worldwide each year. This contributed to 5.3% of all fatalities. Alcohol provides roughly 5.1% of the global burden of disease and injury, measured in Disability Adjusted Life Years (DALYs) (55). As per “Magnitude of Substance Use in India, 2019” report-alcohol is the most common substance used followed by cannabis and opioids. The prevalence of alcohol use is 4.6%, with male: female ratio being 17:1, followed by cannabis at 2.8% and opioids at 2.1%. In regard to harmful and dependent use, 19% of alcohol users use it in dependent pattern, whereas 0.25% of cannabis users use it in dependent pattern (1). Opioid use is reported in 2.1% of the country's population, with heroin use being highest at 1.14% percent followed by pharmaceutical opioids at 0.96% and opium at 0.52%. Regarding the pattern of use, dependent use is highest among users. The prevalence of opioid use in India is three times the global average (56). According to a study conducted by the National Commission for Protection of Child Rights, cannabis and inhalants are the next most popular types of substance abuse among teenagers, apart from alcohol and tobacco. Research indicated that the average age of nicotine use was 12 years old, and another study found that 46% of youths living in slums started using liquor, cannabis, and smokeless tobacco at age 12 or younger (57). For older males, alcohol consumption rates were 8% in Bihar and 5% in Uttar Pradesh, while younger boys were found to use alcohol in Bihar at a rate of 2%. In comparison to younger girls (10–14 years; 1% each in both states) and older, unmarried females (2% in each state), married older girls (15–19 years) were found to consume more tobacco (2% in Bihar and 5% in Uttar Pradesh). The proportion of unmarried girls abusing drugs was 0.1%, compared to none of the married older (15–19-year-old) and unmarried younger (10–14-year-old) girls and boys who had ever used drugs.

### Accidents (drowning and accidents)

#### Drowning

According to estimates, 236,000 individuals globally perished from drowning in 2019, making it a serious public health issue. In 2019, injuries made up nearly 8% of all fatalities worldwide. Seven percent of all injury-related deaths are caused by drowning. A 2019 Lancet analysis on estimates of “healthy life” lost in India projected that there were ~62,000 drowning deaths in 2017 (58).

#### Road accidents

As per ILO estimates, around 2.3 million men and women die each year from occupational injuries or accidents, or more than 6,000 people per day. Every year, nearly 160 million cases of occupational injuries and 340 million workplace accidents occur worldwide (59). Referring to a World Bank study, despite owning only 1% of all vehicles, India accounts for 11% of all road deaths worldwide, the greatest concentration in the world. Over 4.5 lakh road accidents occur in the country each year, with 1.5 lakh persons killed (50). According to the Accidental Deaths & Suicides in India 2020, a total of 354,796 “road accident” instances were registered in 2020, resulting in 335,050 injuries and 133,201 fatalities. In comparison to 2019, the number of unintentional deaths (per lakh of the population) has dropped in 2020. In the year 2020, a total of 374,397 people lost their life due to accident (60).

### Discussion

NCDs are complex, diverse, and significantly influence the health of people in varied ways. There is a need for more comprehensive, holistic and strategic approach for mitigating the impact of non-communicable diseases. We have to think beyond what is being practiced for the past half century pertaining to NCD research, treatment and prevention. Rather than treating the non-communicable diseases, more emphasis should be given to improve the conditions or determinants which lead to the emergence of non-communicable diseases, which hitherto have been largely ignored as global health priorities (10). Besides the major non-communicable diseases including cardiovascular diseases, stroke, cancer, diabetes, and chronic respiratory illness, public health research should accelerate the efforts for the recognition of neglected NCDs as a global health priority.

Some of the common neglected NCDs include gout, asthma, chronic kidney disease, alzheimer's and dementia, sickle cell anemia, alcohol and substance abuse, road accidents, drowning, etc. NNCDs are very important which need urgent addressal. These are not included in NPCDCS and other health programs. Proposed solution for tackling NNCDs through lifecycle approach is given in Table 1. We only focus on BIG FOURs, neglecting other NCDs, but it is the right time to rethink about NNCDs in the existing health programmes. Sickle Cell anemia could be integrated in the National Program for Control of Cardiovascular diseases, Diabetes, Cancer, and Stroke at the health facility level through genetic counseling to the diabetic mothers by the genetic counselors or pediatricians. Aanganwadi workers under Integrated Child Development Services (ICDS) could be engaged for the identification of the early signs of diabetes and proposing the lifestyle changes. At the sub-center level, ASHA and ANMs could screen the children and adolescents for CKD, and Asthma using Spot test method, strip method, breath test and spirometry after conducting proper training to them. The health awareness programs providing the information on accidents, alcohol and substance abuse could be organized at the schools and educational institute level.

**Table 1:** Proposed solution for tackling NNCDs through existing health programs.

**Setting**	**Method**	**Beneficiary**	**Service provider**	**Program**	**Type of NNCD**
Health facility	Genetic counseling	Diabetic mother/SCD	Genetic counselors	(NPCDCS)	Sickle cell anemia
Health facility	Genotyping of infant	Infant/	Pediatricians	“Rashtriya Bal Swasthya Karyakram” (RBSK)	Sickle cell anemia
Anganwadi center	Early detection of symptoms	Children upto age 6years	Anganwadi workers	Integrated Child Development Services (ICDS)	Sickle cell anemia
Health sub-center or health and wellness center	Spot test method, strip method, glucometer test method, spirometry and breath test	Children and adolescents	ASHAs and ANMs	NPCDCS, Rashtriya Kishor Swasthya Karyakram (RKSK)	CKD, CLD, asthma,
Schools and educational institutions	Identification and screening of high risk factors/behaviors and blood sugar	High risk children	Health and wellness ambassadors	School health programs	Accidents, alcohol and substance abuse
Community and national level	Community Based Assessment Checklist (CBAC) form	>30 years, at high risk and with a family history of metabolic syndrome	ASHAs, ANMs, Medical officers	NPCDCS, NPHCE	Gout, asthma, Alzheimer's and dementia

There is very limited evidence available for Gout in India. General practitioners typically treat gout, and only a small percentage of patients may see rheumatologists. Therefore, along with patient education and training, the urgent need of the hour is for the training of physicians, orthopedic, and physiatrists. Chronic liver disorders (CLDs) are common in India somewhat presented late in the clinical course, most frequently after decompensation has begun. This poses a challenge for early treatment and overburden healthcare resources.

These neglected NCDs may be integrated within the ongoing/existing national health programs being implemented at the state and district level. There is a need for coordination, integration, and convergence of efforts in dealing with the above mentioned neglected NCDs. The NHM's primary tactics were applied in the development of the health promotion model in two districts of Punjab and Haryana, with an emphasis on integration, convergence, and the best use of available human and financial resources (61).

The Ministry of Health &Family Welfare, Government of India has been advocating for joint collaboration between various initiatives wherein activities can be planned and carried out, within the framework of the existing health system. For example, the National TB/HIV framework (2013), Pilot project on “Integration of AYUSH with NPCDCS” (2016), National Framework for Joint TB-Tobacco Collaborative Activities (2017), National framework for joint TB-Diabetes collaborative activities (2017), Tribal TB initiative (2021) (62). Besides, Reproductive, Maternal, Newborn, Child and Adolescent Health (antenatal care under PPTCT), Hepatitis B and Hepatitis C (National Viral Hepatitis Control Program) are also linked under HIV sentinel surveillance (63). Chronic Kidney Disease (CKD) guidelines has been included under NPCDCS. Pilot intervention has been initiated for the prevention and control of Rheumatic Fever and Rheumatic Heart Disease under the platforms of NPCDCS and RBSK (Rashtriya Bal Swasthya Karyakram), in three select districts (Gaya—Bihar, Firozabad—Uttar Pradesh and Hoshangabad—Madhya Pradesh). This intervention would be scaled up to other districts in a phased manner (62). The Ministry of Tribal Affairs (MoTA) has launched the Sickle Cell Disease Support Corner to bridge the gap between patients and health care services in tribal areas.

The results from various studies conducted have displayed that such integrative strategies have shown advantage in terms of better cross-referral, improved retention of patients, timely initiation of treatment, and improved survival (64). In recent years, Europe, USA and Caribbean healthcare have adopted universal screening for SCD and where they provide comprehensive care for affected individuals as a result, mortality is now rare among the children born with SCD where the majority of affected children can expect to live a relatively normal life into their 40s and 50s (15–17). A study conducted in India reported that the integration of tobacco cessation interventions into a framework of routine TB care was feasible, provided individual patient benefits and was well-accepted by providers. It further suggested that from a broader health systems perspective, similar initiatives for the integration of tobacco cessation into other relevant national programs should be considered (65). The concept of integration carries forwards the notions of equity, sustainability and community participation. Integration of these neglected NCDs into other NCD control programs should be seen as an opportunity under the health system framework in national policies and health programs.

## Conclusion and recommendations

The screening and prevention of neglected non-communicable diseases need to be strengthened. Experts from public health must reconsider these “forgotten NCDs” on a global and national level. We must investigate the clinical effects of these conditions as well as the consequences of ignoring their medical importance and economic burden on the nations (direct and indirect). With an objective to tackle these neglected non-communicable illnesses within a global NCD program, we need to advise on how they should be prioritized within the global disease burden hierarchy. This will enable us to reduce the global disease burden, minimize DALY, and improve QALY.

## Author contributions

All authors listed have contributed to the article equally and approved it for publication.
